# Protective Effect against Neosporosis Induced by Intranasal Immunization with *Neospora caninum* Membrane Antigens Plus Carbomer-Based Adjuvant

**DOI:** 10.3390/vaccines10060925

**Published:** 2022-06-10

**Authors:** Alexandra Correia, Pedro Alves, Ricardo Fróis-Martins, Luzia Teixeira, Manuel Vilanova

**Affiliations:** 1 ICBAS—Instituto de Ciências Biomédicas de Abel Salazar, Universidade do Porto, Rua Jorge Viterbo Ferreira, 4050-313 Porto, Portugal; alexandra.correia@ibmc.up.pt (A.C.); pedro.lc.alves@gmail.com (P.A.); 2 I3S—Instituto de Investigação e Inovação em Saúde, Universidade do Porto, Rua Alfredo Allen, 4200-135 Porto, Portugal; 3 Immunology Section, Vetsuisse Faculty, University of Zurich, Winterthurerstrasse 266a, 8057 Zurich, Switzerland; raf.martins@campus.fct.unl.pt; 4 Institute of Experimental Immunology, University of Zurich, Winterthurerstrasse 190, 8057 Zurich, Switzerland; 5 UMIB—Unidade Multidisciplinar de Investigação Biomédica, ICBAS—Instituto de Ciências Biomédicas de Abel Salazar, Universidade do Porto, Rua de Jorge Viterbo Ferreira, 4050-313 Porto, Portugal; lmteixeira@icbas.up.pt; 6 ITR—Laboratory for Integrative and Translational Research in Population Health, 4050-290 Porto, Portugal

**Keywords:** *Neospora caninum*, vaccine, adjuvant, interferon-γ, interleukin-17

## Abstract

*Neospora caninum* is an obligate intracellular protozoan responsible for abortion and stillbirths in cattle. We previously developed a mucosal vaccination approach using *N. caninum* membrane proteins and CpG adjuvant that conferred long-term protection against neosporosis in mice. Here, we have extended this approach by alternatively using the carbomer-based adjuvant Carbigen™ in the immunizing preparation. Immunized mice presented higher proportions and numbers of memory CD4^+^ and CD8^+^ T cells. Stimulation of spleen, lungs and liver leukocytes with parasite antigens induced a marked production of IFN-γ and IL-17A and, less markedly, IL-4. This balanced response was also evident in that both parasite-specific IgG1 and IgG2c were raised by immunization, together with specific intestinal IgA. Upon intraperitoneal infection with *N. caninum*, immunized mice presented lower parasitic burdens than sham-immunized controls. In the infected immunized mice, memory CD4^+^ T cells predominantly expressed T-bet and RORγt, and CD8^+^ T cells expressing T-bet were found increased. While spleen, lungs and liver leukocytes of both immunized and sham-immunized infected animals produced high amounts of IFN-γ, only the cells from immunized mice responded with high IL-17A production. Since in cattle both IFN-γ and IL-17A have been associated with protective mechanisms against *N. caninum* infection, the elicited cytokine profile obtained using Carbigen^TM^ as adjuvant indicates that it could be worth exploring for bovine neosporosis vaccination.

## 1. Introduction

The apicomplexan *Neospora caninum* is an obligatory intracellular coccidian parasite. Domestic and wild canids are the definitive hosts of *N. caninum* while farm ruminants are the economically important definitive hosts of this parasite, which has been also isolated from horses, deer and buffaloes [1]. *N. caninum* is a major causative agent of abortion and stillbirths in cattle [2]. Neosporosis is prevalent worldwide [1] and inflicts high economic losses to dairy cattle systems [3,4], which have been estimated above one billion US dollars per annum [5]. A recent meta-analysis study estimated that the highest prevalence of bovine neosporosis occurs in North, Central and South America, followed by Asia, Europe, and Oceania [6]. The highly effective vertical transmission of *N. caninum* in cattle significantly contributes to the burden of disease [7,8]. Several control measures could be considered to manage neosporosis, ranging from “doing nothing” to “test-and-cull” and chemotherapeutical approaches [9,10]. However, vaccination is still considered the most cost-effective approach to manage neosporosis, especially in high-prevalence farms [11]. Currently, no commercial vaccine is available to prevent or treat *N. caninum* infection, despite several approaches that have been attempted to achieve it [12]. A Th1-type response mediated by the IL-12/IFN-γ axis has been considered the prototypic protective immune response in *N. caninum*-infected hosts [13]. Indeed, numerous studies in different host species have associated the production of IFN-γ to protection against neosporosis [13,14].

Studies in mice showed the preponderant role of IFN-γ produced by CD4^+^ and CD8^+^ T cells in protection against this parasitic infection [15,16]. Moreover, a direct effect of this cytokine in limiting parasite multiplication in bovine cells has also been demonstrated [17]. Stimulation of nitric oxide production and of immunity-related GTPases and guanyl-ate-binding proteins have been highlighted as effector mechanisms triggered by IFN-γ [16,18,19,20,21,22]. More recently, a protective role of IL-17A against *N. caninum* infection in the bovine model has also been suggested [23,24]. However, a beneficial or deleterious role of this cytokine in the course of infection is yet uncertain [25,26].

Considering the preponderant role of IFN-γ in host protection against neosporosis, several vaccination strategies have stressed the induction of the production of this cytokine through selected antigen and adjuvants [27,28,29,30,31,32]. We have previously developed a mucosal immunization approach aimed at preventing *N. caninum* infection that used an extract of the parasite membrane antigens together with CpG oligodeoxynucleotide adjuvant to promote Th-1-type and humoral immunity [33]. This approach proved highly effective in protecting against *N. caninum* infection in the long term [34]. Here, we extended our previous work by using the same mucosal immunization approach, using instead the carbomer-based adjuvant Carbigen™ in the immunizing preparation. The low cost of this adjuvant, as compared to previously used CpG, plus its suitability to be used in veterinary immunization [35,36], prompted us to assess its effectiveness in a murine model of neosporosis established intraperitoneally (i.p.). Our results highlighted immune parameters resulting from the immunization approach used here that make Carbigen^TM^ a promising adjuvant to be similarly assessed in the bovine model.

## 2. Materials and Methods

### 2.1. Mice

Female C57BL/6 mice were purchased from Charles River (Barcelona, Spain) and bred under specific pathogen-free conditions at the animal facilities of Instituto de Ciências Biomédicas Abel Salazar (ICBAS). Housing and nesting materials were provided as enrichment. Experiments were approved by the institutional board responsible for animal welfare at ICBAS (ORBEA, document 109/2015) and by the competent national authority (DGAV, document 0421/000/000/2016).

### 2.2. Growth of Parasites and Preparation of Tachyzoite Lysates and Cell Membrane Extracts

*N. caninum* tachyzoites (Nc1 isolate) were serially passaged in VERO cell cultures to obtain free parasitic forms using a previously described methodology [37]. Parasite concentration was determined in cell suspensions using a hemocytometer. Whole parasite sonicates and *N. caninum* antigen extracts enriched in parasite membrane proteins (NcMP) were prepared accordingly to previously described methodology [33]. Briefly, frozen tachyzoites were suspended in PBS containing 0.75% Triton X-14 and the organic phase was precipitated with 4 volumes of absolute ethanol. After centrifugation, the protein extract was air dried and suspended in PBS. Protein concentration was determined using the Pierce™ BCA Protein Assay Kit (Thermo Fisher Scientific, Rockford, IL, USA) according to the manufacturer’s instructions.

### 2.3. Immunizations and Tissue Sample Collection

Mice, 8–10 weeks-old, randomly distributed into 2 groups, were immunized intranasally (i.n.) with 15 μL of PBS containing 10% of carbomer-based (Carbopol 934P) adjuvant suspension Carbigen^TM^ (MVP adjuvants, Omaha, NE, USA) (CARB group) or with PBS containing 30 μg of NcMP plus 10% Carbigen^TM^ (NcMP/CARB group). A boost immunization was done three weeks later. Three weeks after the boost immunization, mice were either sacrificed by cervical dislocation upon isoflurane anesthesia for organ collection or i.p. challenged with 1 × 10^7^ *N. caninum* tachyzoites. Infected mice were similarly sacrificed seven days after infection. Spleens, lungs and liver were collected under aseptic conditions to analyze the elicited immune response. Brain, liver and lungs samples were collected and stored frozen at −20 °C until processing for DNA extraction. Serum was prepared from clotted blood samples after centrifugation at 10,000× *g* for 15 min at 4 °C. Serum was then transferred to new tubes and stored frozen at −20 °C for antibody quantification. Intestinal lavage fluids were prepared as follows: PBS with protease inhibitors (Mini Complete, Roche, Basel, Switzerland) was consecutively passed through the small intestine lumen and then centrifuged at 4500× *g* for 15 min at 4 °C. The supernatant was collected and centrifuged at 10,000× *g* for 1 h at 4 °C, and the resulting supernatant was collected and kept frozen at −20 °C for subsequent antibody detection.

### 2.4. Antibody Detection

Titers of NcMP-specific serum IgG1 and IgG2c and of IgA in intestinal lavage fluids were quantified by ELISA, using respective alkaline phosphatase-coupled goat anti-mouse antibodies (all from Southern Biotechnology Associates, Birmingham, AL, USA) by a previously described methodology [33].

### 2.5. In Vitro Cell Cultures and Cytokine Detection

For cytokine production assessment, spleens were mechanically homogenized in Hanks’ balanced salt solution (HBSS) (Sigma) and passed through 100 µm cell strainers (BD Falcon, Franklin Lakes, NJ, USA). Ammonium–Chloride–Potassium Lysing Buffer (ACK) was added to lyse red blood cells. Remaining cells were washed in HBSS and suspended in RPMI-1640 medium supplemented with 10% Fetal Bovine Serum (FBS), HEPES (10 mM), penicillin (200 IU/mL) and streptomycin (200 µg/mL) (all from Sigma, Burlington, MA, USA) (RPMI), and β-mercaptoethanol (0.05 µM) (Merk, Darmstad, Germany). The lungs and livers were cut in small pieces and placed in RPMI containing 2 mg/mL Collagenase D (Roche, Basel, Switzerland) for 45 min at 37 °C in a water bath with agitation. Then, samples were homogenized and passed through 100 µm pore cell strainers. Liver leukocytes were isolated through density gradient centrifugation using 33% Percoll (GE Healthcare, Chicago, IL, USA) solution in PBS for 12 min at room temperature at 750× *g* with minimal brake. Cells from lungs and liver were washed twice with HBSS 2% FBS before being resuspended in 500 µL supplemented RPMI. In between washes, ACK was added for red blood cell lysis. Spleen, liver and lung cell concentrations were adjusted, and cells were plated (2 × 10^5^/well) in round bottom 96-well plates (Nunc, Roskilde, Denmark) and were left unstimulated or stimulated with *N. caninum* sonicates (25 µg/mL) for 3 days at 37 °C and 5% CO_2_. Then, supernatants were collected for IFN-γ, IL-4, and IL-17A cytokine measurements by ELISA, using respective eBioscience™ Mouse ELISA Ready-SET-Go!™ kits according to manufacturer’ instructions.

### 2.6. Flow Cytometry Analysis

The following mAbs were used for surface antigen staining: anti-mouse CD3 eFluor 506-conjugate (clone 17A2), anti-mouse CD4 eFluor 450-conjugate (clone RM4–5), anti-mouse CD44 PE-Cy7-conjugate (clone IM7), anti-mouse CD62L PE-conjugate (clone MEL-14) (all from eBioscience, San Diego, CA, USA) and anti-mouse CD8 FITC-conjugate (clone 53-6.7) (BioLegend, San Diego, CA, USA). Cell viability was assessed using APCeFluor 780 Fixable viability dye (FVD; eBioscience). Firstly, cells were stained with FVD and incubated 30 min on ice. After washing with PBS, antibodies specific for surface markers were added to the cells and incubated for 25 min on ice, protected from light. After washing with FACS buffer (10 mM Sodium Azide, 2% FBS in PBS), cells were fixed with Foxp3 Fixation/ Permeabilization solution (eBioscience) and permeabilized using Foxp3 Permeabilization Buffer (eBioscience). For Fcγ receptor nonspecific binding, cells were preincubated with anti-mouse CD16/CD32 (BioLegend) before staining with anti-mouse T-bet PerCP-conjugate (clone eBio4B10), anti-mouse RORγt APC-conjugate (clone B2D), anti-mouse GATA-3 AlexaFluor488-conjugate (clone TWAJ) and anti-granzyme B APC-conjugate (clone NGZB) (all from eBioscience). Fluorescence minus one staining were done for gating purposes. Data were acquired in a BD FACSCanto™ II cytometer (BD Biosciences, Franklin Lakes, NJ, USA) and analyzed using FlowJo version 10.8.1 (Tree Star inc., Ashland, OR, USA). Used gating strategies are shown in Figures S1 and S2.

### 2.7. DNA Extraction and Real-Time PCR Analysis

DNA was extracted from the brain, liver and lungs of infected mice, as previously described [38]. Parasite burden was assessed by quantitative real-time PCR (qPCR) using the primers NcA 5′-GCTACCAACTCCCTCGGTT-3′ and NcS 5′-GTTGCTCTGCTGACGTGTCG-3′, the TaqMan fluorescent probe FAM-CCCGTTCACACACTATAGTCACAAACAAAA-BBQ (all from TIB Molbiol GmbH, Berlin, Germany) and NZY qPCR Probe Master Mix (Nzytech, Lisbon, Portugal). Samples were run in a Corbett rotor gene 6000 system (Corbett Life Science, Sydney, NSW, Australia), according to previously described methods [16]. In all runs, parasite burden was determined by interpolation of a standard curve performed with DNA isolated from *N. caninum* tachyzoites, ranging from 10 to 1 × 10^−4^ ng of parasitic DNA (2 to 2 × 10^5^ parasites), included in each run. Data were analyzed in the Rotor gene 6000 software v1.7 (Corbett Life Science) and expressed as log10 parasites per mg of DNA.

### 2.8. Statistical Analysis

Statistical analyses were performed using GraphPad prism version 9.0 (GraphPad Software, Inc., La Jolla, CA, USA). Scatter dot graphs with bars represent mean and individual values. Test for normal distribution was assessed with Shapiro–Wilk test and Kolmogorov–Smirnov test. When passing normality test data were analyzed using unpaired Student’s *t*-test. Otherwise, Mann–Whitney test was used, as indicated in figure legends.

## 3. Results

### 3.1. T Cell Response to N. caninum Antigens Induced by Immunization

**Figure 1:**
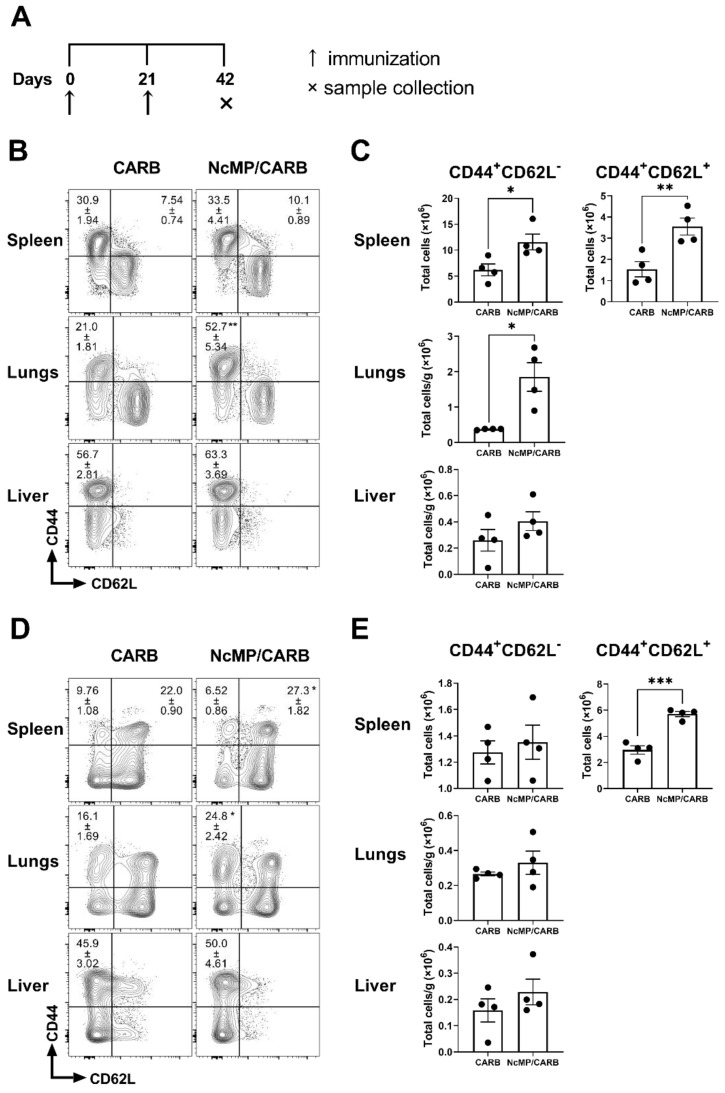
Memory phenotype of CD4^+^ and CD8^+^ T cells. Timeline of immunization and sample collection (**A**). Representative contour plot analysis of gated CD4^+^ (**B**) and CD8^+^ (**D**) T cells expressing CD44 and CD62L in the indicated organs, 21 days after the boost immunization. Numbers within contour plots correspond to mean percentage values ± SEM for the particular quadrant region in the respective sham-immunized (CARB) or immunized (NcMp/CARB) groups, as indicated. Numbers of CD4^+^ (**C**) and CD8^+^ (**E**) T cells presenting the cell surface phenotypes CD44^+^CD62L^−^ and CD44^+^CD62L^+^, as indicated, in the analyzed organs of CARB and NcMp/CARB groups. In panels (**C**,**E**), bars represent means, and each symbol (black circle) represents an individual mouse ± SEM. Results are of one representative experiment out of two independent experiments that yielded concordant results (*n* = 4 group; * *p* < 0.05; ** *p* < 0.01; *** *p* < 0.001, unpaired *t*-test).

Three weeks after the boost immunization, mice of the NcMP/CARB group presented higher numbers of spleen CD4^+^ and CD8^+^ T cells displaying a CD44^+^CD62L^+^ phenotype (Figure 1), characteristic of central memory T cells (T_CM_) [39], as compared to the control CARB group. In the spleen and lungs, higher numbers of CD4^+^ effector memory (CD44^+^CD62L^−^) T cells (T_EM_) were also found.

**Figure 2:**
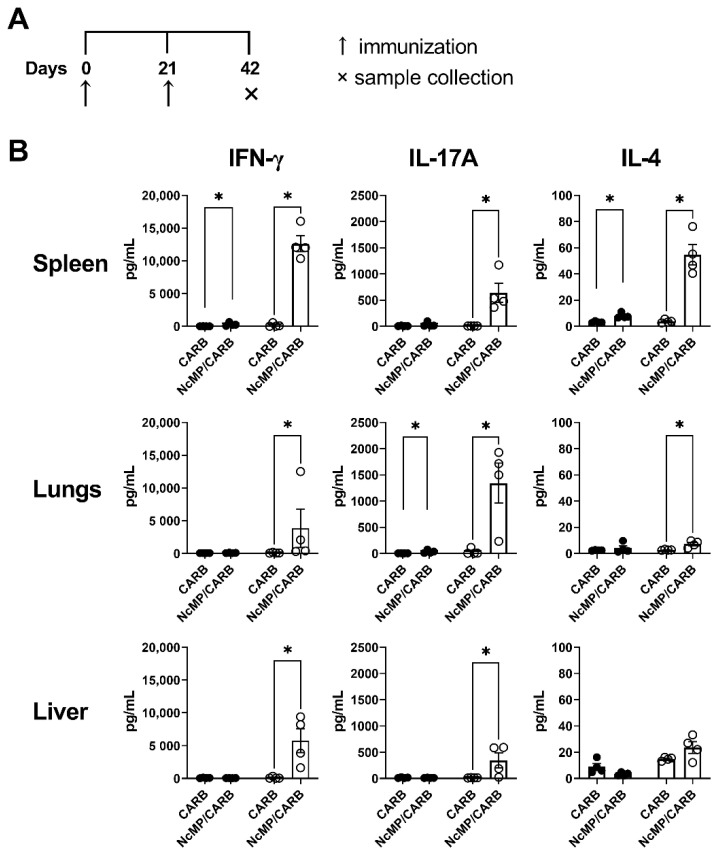
Analysis of cytokine production. Timeline of immunization and sample collection (**A**). IFN-γ, IL-17A and IL-4 concentration (**B**) in the supernatants of leukocyte cell cultures unstimulated (closed circles) or stimulated (open circles) for 3 days with *N. caninum* whole parasite antigen sonicates. Cells were isolated from the spleen, lungs and liver of mice from NcMP/CARB or control CARB groups, as indicated, 21 days after the boost immunization. Each symbol represents an individual mouse; bars correspond to the mean value in each group ± SEM. Results are from one representative experiment out of two independent experiments that yielded concordant results. (*n* = 4 group; * *p* < 0.05, Mann–Whitney.)

Spleen, liver and lung leukocytes from mice of the NcMP/CARB group stimulated ex vivo with *N. caninum* whole parasite antigen sonicates responded with higher production of IFN-γ and IL-17A, comparatively to similarly stimulated cells obtained from the CARB group mice (Figure 2). Production of IL-4 was also elevated in the cultures of spleen and lung cells of the NcMP/CARB group that was nevertheless detected at lower levels.

**Figure 3:**
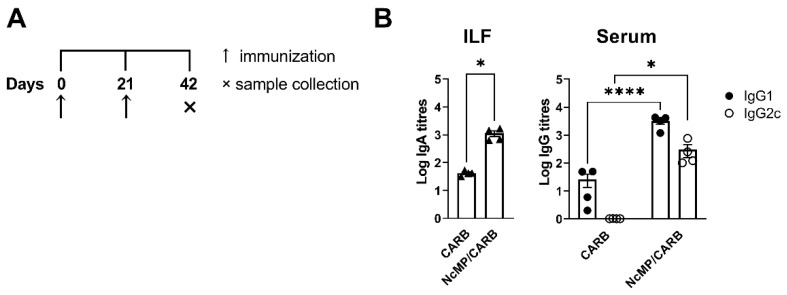
Analysis of *N. caninum*-specific antibodies. Timeline of immunization and sample collection (**A**). Titers of intestinal lavage fluid (ILF) IgA and serum (Serum) IgG1 and IgG2c (**B**), as indicated, were determined by ELISA in samples collected, on the indicated day, from mice of CARB and NcMP/CARB groups. Data is presented as log_10_ of the antibody titers (*n* = 4 per group). Each symbol (triangles and circles) represents an individual mouse. Bars correspond to the mean value in each group ± SEM. Results are of one representative experiment out of two independent experiments that yielded concordant results (*n* = 4 group; * *p* < 0.05; **** *p* < 0.0001, Mann–Whitney).

Parasite-specific serum IgG1 and IgG2c and intestinal lavage fluid IgA levels were elevated in the NcMP/CARB group as compared to controls (Figure 3). These results altogether demonstrated that the used immunization approach induced a parasite-specific response mediated by T and B cells, characterized by a mixed cytokine profile that was mainly mediated by proinflammatory cytokines IFN-γ and IL-17A, but also involving IL-4 production.

### 3.2. Parasitic Burden in Immunized Mice

**Figure 4:**
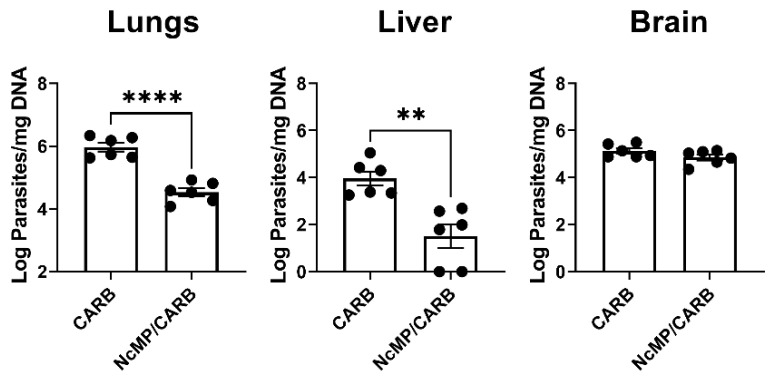
Protective effect of i.n. immunization with NcMP plus Carbigen^TM^ adjuvant against i.p. *N. caninum* infection. Parasitic load was determined through qPCR 7 days upon i.p. challenge with 1 × 10^7^ *N. caninum* tachyzoites in immunized (NcMP/CARB) or sham-immunized (CARB) mice. Data are presented as log_10_ parasites per mg of total DNA. Results are of one representative experiment out of two independent experiments that yielded concordant results. Each symbol (black circle) represents an individual mouse. Bars correspond to the mean value in each group; (*n* = 6 group; ** *p* < 0.01; **** *p* < 0.0001, unpaired *t*-test).

To assess whether the induced response could protect against *N. caninum* infection, mice of the CARB and NcMP/CARB groups were infected i.p. with 1 × 10^7^ *N. caninum* tachyzoites three weeks after the boost immunization. As shown in Figure 4, 7 days upon infection, the NcMP/CARB group presented lower parasitic burdens in the lungs and liver than the sham-immunized CARB group. No significant difference was found in the brain parasitic burden. These results show that the used immunization procedure conferred partial protection against the disseminated *N. caninum* infection established i.p.

### 3.3. Parasite-Specific Antibody Levels in Infected Immunized and Nonimmunized Mice

**Figure 5:**
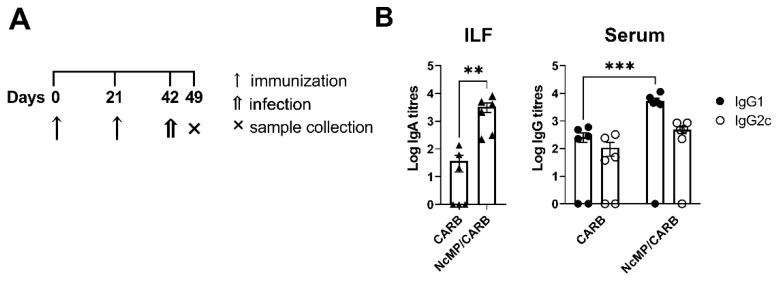
Analysis of *N. caninum*-specific antibodies in infected mice. Timeline of immunization, infection and sample collection (**A**). Titers of intestinal lavage fluid (ILF) IgA and serum (Serum) IgG1 and IgG2c (**B**), as indicated, were determined by ELISA in samples collected 7 days after *N. caninum* i.p. infection from mice previously immunized (NcMP/CARB) or sham-immunized (CARB). Data is presented as log_10_ of the antibody titers. Each symbol (triangles and circles) represents an individual mouse. Bars correspond to the mean value in each group ± SEM. Results are of one representative experiment out of two independent experiments that yielded concordant results. (*n* = 6 group; ** *p* < 0.01; *** *p* < 0.001, Mann–Whitney.)

As shown in Figure 5, 7 days upon infection the levels of parasite-specific serum IgG1 and IgG2c antibodies were found elevated in the NcMP/CARB mouse group comparatively to the CARB group. As observed prior to infection, IgG1 reached higher titers than IgG2c in the immunized group. Parasite-specific IgA levels were also found elevated in intestinal lavage fluids collected from the mice of the NcMP/CARB group as compared to the CARB group. These results showed that the production of parasite-specific antibodies induced by immunization was effective both in the intestinal mucosa and systemically.

### 3.4. Differentiation of T cells Induced in Immunized Mice Infected with N. caninum

**Figure 6:**
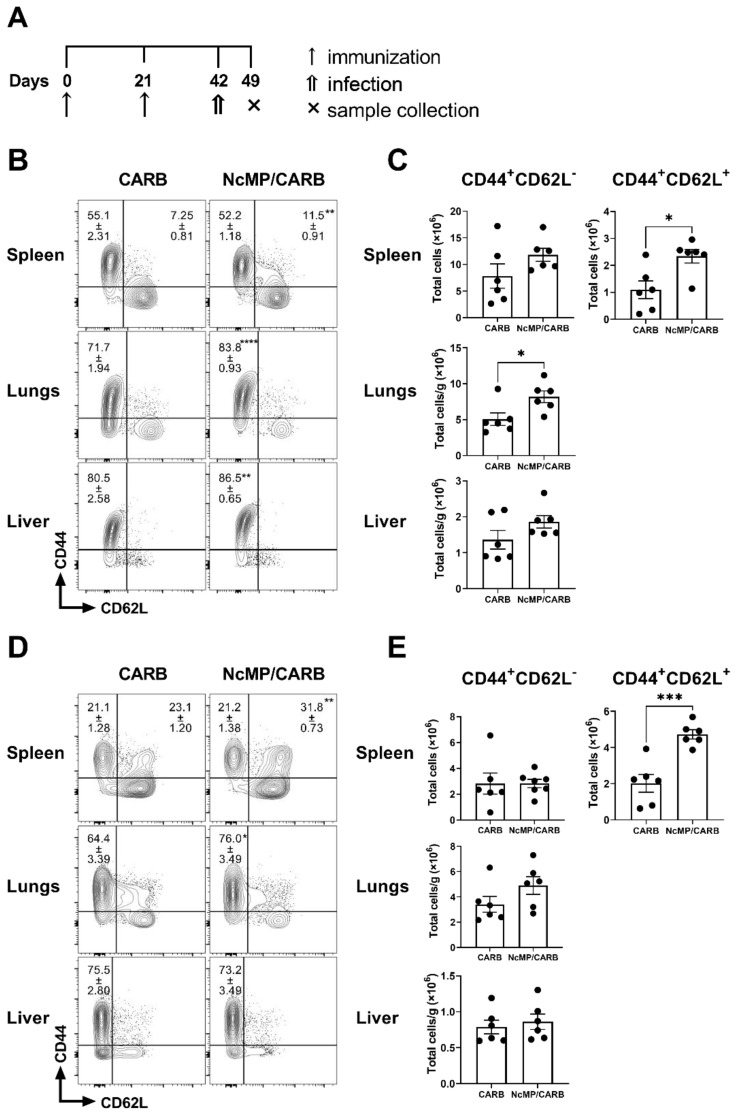
Memory phenotype of CD4^+^ and CD8^+^ T cells in infected mice. Timeline of immunization, infection and sample collection (**A**). Representative contour plot analysis of gated CD4^+^ (**B**) and CD8^+^ (**D**) T cells expressing CD44 and CD62L in the indicated organs. Numbers within contour plots correspond to mean percentage values ± SEM for the particular quadrant region in the respective sham-immunized (CARB) or immunized (NcMP/CARB) groups, as indicated. Numbers of CD4^+^ (**C**) and CD8^+^ (**E**) T cells presenting the cell surface phenotypes CD44^+^CD62L^−^ and CD44^+^CD62L^+^, as indicated, in the analyzed organs of CARB and NcMP/CARB groups, 7 days upon *N. caninum* i.p. infection. In panels (**C**,**E**), bars represent means ± SEM. Each symbol (black circle) represents an individual mouse. Results are from one experiment representative of two independent experiments that yielded concordant results (*n* = 6 group; * *p* < 0.05; ** *p* < 0.01; *** *p* < 0.001; **** *p* < 0.0001, unpaired *t*-test).

As shown in Figure 6B,C, 7 days upon infection, the NcMP/CARB group presented higher proportions and numbers of CD4^+^ T_CM_ cells in the spleen. Immunized mice presented higher proportions of CD4^+^ T_EM_ cells in the lungs and liver. T_EM_ cell numbers were also found elevated in the lungs of the immunized mice as compared to controls. CD8^+^ T_EM_ cells were found at higher proportions in the lungs of NcMP/CARB group mice (Figure 6D) while in the spleen CD8^+^ T_CM_ cell proportions and numbers were both found elevated as compared to control group (Figure 6D,E). As expected upon infection, T_EM_ cells predominated in the infected organs.

**Figure 7:**
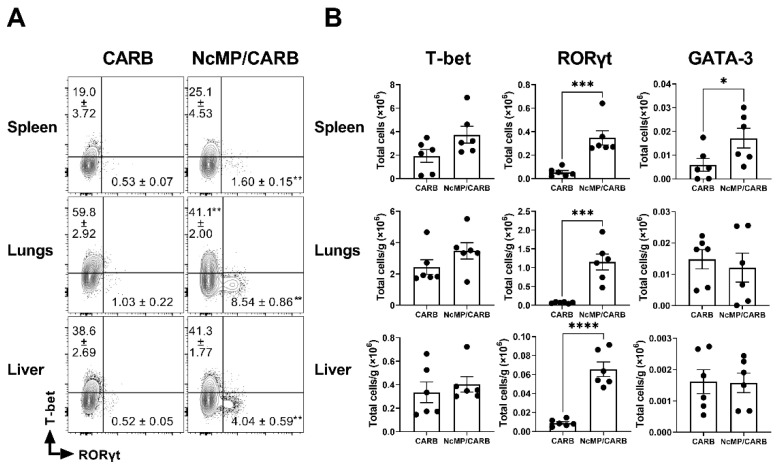
Expression of T-bet, RORγt and GATA-3 in CD4^+^CD44^+^ T cells collected from infected mice. (**A**) Representative contour plots of T-bet and RORγt expression in CD4^+^ CD44^+^ T cells in the indicated organs of sham-immunized (CARB) or immunized (NcMP/CARB) mice, as indicated, 7 days after i.p. challenged with 1 × 10^7^ *N. caninum* tachyzoites. Numbers within contour plots correspond to mean percentage values ± SEM of cells in the respective analysis regions. (**B**) Numbers of CD4^+^ CD44^+^ T cells expressing T-bet, RORγt and GATA-3 in the indicated organs of infected mice of NcMP/CARB and CARB mouse groups. Each symbol (black circle) represents an individual mouse. Results are representative of two independent experiments that yielded concordant results (*n* = 6 group; * *p* < 0.05; ** *p* < 0.01; *** *p* < 0.001; **** *p* < 0.0001, Mann–Whitney).

To determine the type of cellular response induced upon infection in the immunized mice and controls, we evaluated the expression of the transcription factors T-bet, GATA-3 and RORγt, respectively, associated with Th1, Th2 and Th17 cell populations [40] in antigen-experienced (CD44^+^) CD4^+^ T cells. As shown in Figure 7, immunized mice clearly presented higher proportions and numbers of activated/memory CD4^+^ T cells expressing RORγt in all analyzed organs. In the spleen, GATA-3-expressing CD4^+^ T cells were also increased in the NcMP/CARB group. Splenic T-bet-expressing CD4^+^ T cells were detected at elevated numbers and proportions in immunized mice, although not reaching statistical significance. Similar results were found when evaluating the expression of these transcription factors in gated CD4^+^ T_EM_ cells (Figure S3).

**Figure 8:**
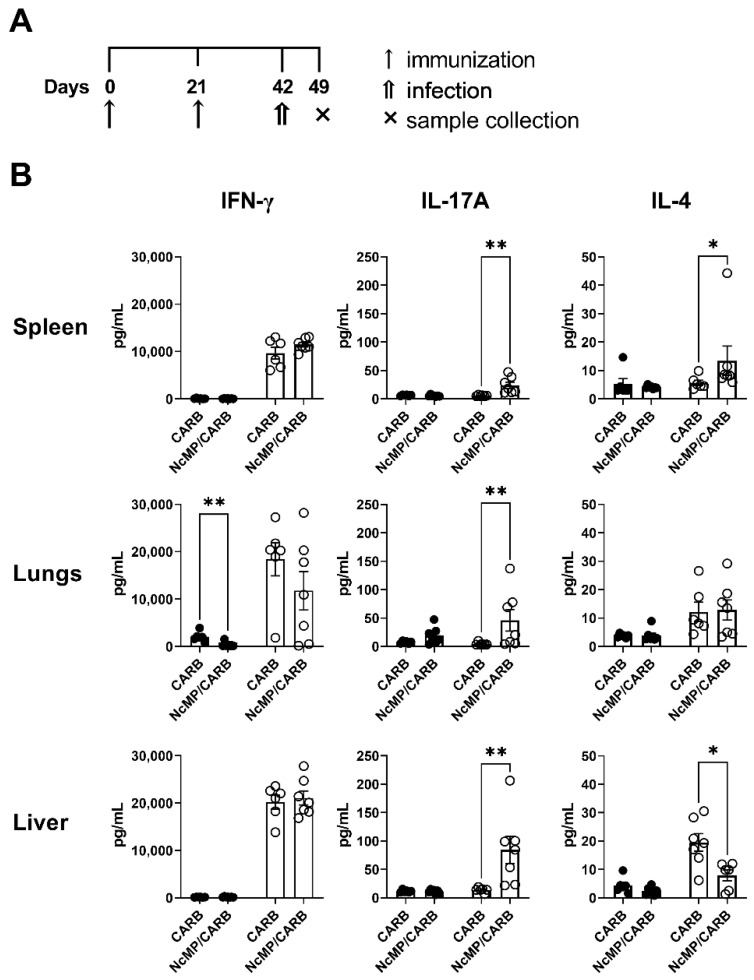
Analysis of cytokine production. Timeline of immunization, infection and sample collection (**A**). IFN-γ, IL-4 and IL-17A concentration (**B**) in the supernatants of splenocyte cell cultures unstimulated (closed symbols) or stimulated (open symbols) for 3 days with *N. caninum* whole parasite antigen sonicates. Cells were isolated from the spleen, lungs and liver of mice from NcMP/CARB or control CARB groups, as indicated, 7 days after the i.p. challenge with 1 × 10^7^ *N. caninum* tachyzoites. Each symbol (black and white circles) represents an individual mouse; bars correspond to the mean value in each group ± SEM. Results are representative of two independent experiments that yielded concordant results (*n* = 6 group; * *p* < 0.05; ** *p* < 0.01, Mann–Whitney).

Culture supernatants of *N. caninum* antigen-stimulated NCMP/CARB group lung leukocytes collected 7 days upon *N. caninum* infection had higher levels of IL-17A (Figure 8), while the levels of IFN-γ and IL-4 did not differ from controls. A bias towards IL-17A production in response to *N. caninum* antigens was also detected in cultures of liver leukocytes from the NCMP/CARB group. The levels of IFN-γ were also detected elevated in these cultures, however not differing between the NcMP/CARB and CARB groups.

CD8^+^ T cells have been also implicated in the protective immune response to acute neosporosis mediated by IFN-γ [16]. Therefore, the expression of T-bet was also assessed in the spleen, liver and lungs of CD44-expressing CD8^+^ T cells.

**Figure 9:**
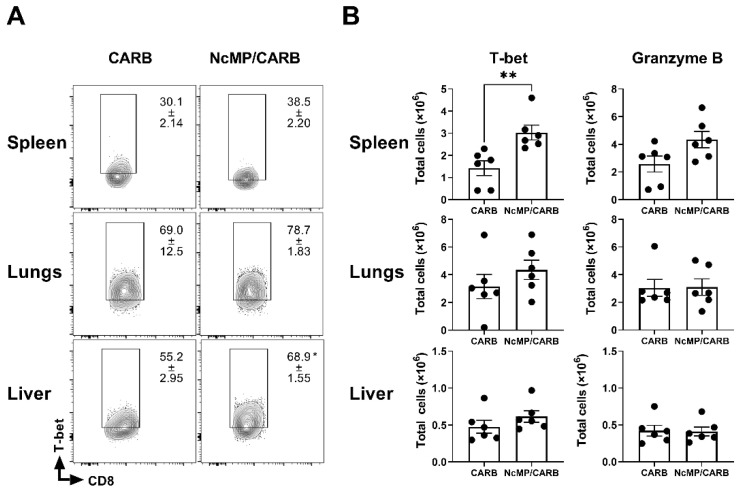
Expression of T-bet and granzyme B in CD8^+^ T cells of infected mice. (**A**) Representative contour plot analysis of CD8^+^ CD44^+^ T cells expressing T-bet in the indicated organs of sham-immunized (CARB) or immunized (NcMp/CARB) mice, as indicated, 7 days after i.p. challenged with 1 × 10^7^ *N. caninum* tachyzoites. Numbers within contour plots correspond to mean percentage values ± SEM of T-bet expressing cells within the CD8^+^ CD44^+^ T cell population. (**B**) Numbers of CD8^+^ CD44^+^ T-bet^+^ and CD8^+^CD44^+^ granzyme B+ cells in the indicated organs of immunized and sham-immunized mice. Each symbol (black circle) represents an individual mouse. Results are representative of two independent experiments that yielded concordant results (*n* = 6 group; * *p* < 0.05; ** *p* < 0.01, unpaired *t*-test).

As shown in Figure 9, higher total numbers of spleen T-bet-expressing CD8^+^CD44^+^ T cells were detected in the *N. caninum* infected NcMP/CARB mouse group than in the control group. Higher proportions, but not total numbers, of T-bet-expressing CD8^+^CD44^+^ T cells were observed in the liver of immunized mice when compared to controls. No such difference was observed between groups in the lungs, further suggesting the strong polarization towards a Th17-type response induced by the intranasal immunization in this organ. The total numbers of granzyme B-expressing cells were not significantly different between mouse groups. The analysis of the expression of T-bet and granzyme B within the CD8^+^ T_EM_ cell compartment revealed similar results (Figure S4). Altogether, these results show that immunization with NcMP plus Carbigen^TM^ induced the differentiation of Th-1 and Th-17 cells and highlight the impact of the local environment in the elicited response.

## 4. Discussion

We have previously reported a mucosal immunization approach to prevent neosporosis based on the intranasal administration of an *N. caninum* membrane antigen extract and CpG adjuvant [33]. This immunization approach was conceived aiming at inducing parasite-specific Th-1-type immunity that is promoted by CpG [41]. It is well established that IL-12 and IFN-γ are key cytokines in host protection from neosporosis, as clearly shown in several genetically deficient murine models [19,42,43]. Using Carbigen^TM^ as the selected adjuvant in the immunization performed here prompted spleen and liver leukocyte cells obtained from the NcMP/CARB group to robustly produce IFN-γ upon *N. caninum* antigen stimulation. This response may contribute to the protective effect against *N. caninum* infection observed in that mouse group and concurs with the generation of T-bet^+^ CD8^+^ T cells induced by immunization. Indeed, using a similar model of infection, we showed that IFN-γ production, rather than cytotoxicity, was the preponderant CD8^+^ T cell effector mechanism counteracting *N. caninum* in the acute phase of infection [16].

T-bet expression in CD8^+^ T cells has been associated with effector rather than memory cell differentiation [44]. As CD8^+^ T cells have been implicated in IFN-γ-dependent immunity to *N. caninum* [16,45], it will be interesting to assess CD8^+^ T cell function at a longer term in immunized animals. On the other hand, as T-bet has been shown to limit the expression of the inhibitory cell surface receptor programmed cell death protein 1 (PD-1) on CD8^+^ T cells [46], the higher expression of this transcription factor may help sustain the effector function of these lymphocytes in infected immunized hosts.

A marked production of IL-17A was also induced in the immunized animals. While this cytokine is usually associated with immune protection from fungal and bacterial infections [47], it can also play a host protective role in diverse protozoan infections [48], including *N. caninum*-related protozoan *Toxoplasma gondii* [49]. Indeed, IL-17 receptor-deficient mice displayed increased mortality when infected with *T. gondii*, which has been attributed to impaired neutrophil recruitment to infected sites [49]. Moreover, the production of IL-17A has been associated with reduced parasitic burden in mice immunized with *T. gondii* protein ROP13 [50]. Here, IL-17A production was evident in parasite antigen-stimulated leukocyte cells obtained from immunized mice prior or after infection. This is in accordance with the observed increase in CD44^+^CD4^+^ T cells expressing the Th17-associated marker RORγt in immunized mice. Th17 cells have been particularly implicated in the local protective immune response to pulmonary infections caused by bacteria, fungi or viruses [51]. Moreover, these cells have been implicated in protective mechanisms against protozoan infections [52,53]. It would therefore be worth determining whether these cells could be mediating the pulmonary protective effect observed in the NcMP/CARB mouse group, since IL-17-mediated responses in the lungs induced by intranasal immunization have been previously associated with host protection from diverse infections [54,55,56,57]. The intranasal immunization used here also induced *N. caninum* antigen responsive liver leukocyte cells producing IL-17A. Even though local production of this cytokine in that organ may have contributed to the lower parasitic burden, it may also play a regulatory role therein, as previously described in *T. gondii*-infected mice [58]. Although the specific role of this cytokine has not yet been elucidated in the context of *N. caninum* infection, the scarce evidence available indicates that in ruminants, IL-17 may be involved in host protection by mediating parasite elimination [24] or limiting its vertical transmission [23]. These findings support exploring in cattle the immunization strategy addressed here, based on using Carbigen^TM^ adjuvant.

Although we observed here that the used immunization approach conferred partial protection against *N. caninum* infection in the lungs and liver, it did not lead to significantly reduced parasitic burden in the brain, contrasting previous results obtained using CpG adjuvant in a similar i.n. immunization approach [33]. A less exacerbated Th1-type polarization in the immune response induced by the adjuvant used here may have accounted for the absence of a protective effect in the brain tissue.

Antibodies have been previously implicated in host protective mechanisms operating against *N. caninum* infection locally at the intestinal mucosa and systemically [33,34,59]. The results obtained here showed the effectiveness of using Carbigen^TM^ adjuvant to raise parasite-specific intestinal IgA and circulatory IgG levels, which might, respectively, counteract the parasite entry into the host at the intestinal tract and parasite dissemination within the host. The IgG antibodies raised by the immunization presented a mixed IgG1 and IgG2c isotype profile that was detected prior to and after infection, in accordance with the detected production of IL-4 and IFN-γ in the culture supernatants of the antigen-stimulated leukocytes isolated from the NcMP/CARB mice. This cytokine and antibody profile indicate that a balanced immune response was induced by the immunization. A balanced Th1/Th2 response has been considered beneficial in the course of neosporosis in pregnant mouse models by conferring protection against the parasite and avoiding fetal rejection by limiting deleterious excessive inflammation [25]. Moreover, an antibody profile with higher IgG1 than IgG2 antibodies combined with IFN-γ was previously associated with the reduced vertical transmission of *N. caninum* in cattle [60], which further highlights the potential of considering the Carbigen^TM^ adjuvant in future studies of immunization against neosporosis in cattle. Although the murine model has been rightly considered a valuable experimental tool in preclinical studies in neosporosis [61], species-specific immune mechanisms should be accounted when evaluating immunization approaches to this parasitic disease. Additionally, as the immunization approach used here raised parasite specific antibodies in the gut and a balanced immune response, deemed convenient during pregnancy, it would be interesting to assess this immunizing procedure in animal models where infection could be established via the gastrointestinal tract or through vertical transmission.

## 5. Conclusions

Our results show that intranasal immunization with NcMP as the target antigens plus the Carbigen^TM^ adjuvant promoted a balanced immune response characterized by a preponderant differentiation of RORγt- and T-bet-expressing memory T cells, leading to the predominant production of IFN-γ and IL-17A in response to parasite antigens. In the immunized animals, a lower parasitic burden was detected in the lungs and liver. Although the lack of detected protection in the brain may pose a limitation to this adjuvant, the results reported here indicate that, as previously highlighted by others [24], exploring more in depth the role of IL-17-promoting adjuvants, such as we observed here using Carbigen^TM^, will be nevertheless worth attempting in vaccination approaches to neosporosis.

## Data Availability

The data presented in this study are available on reasonable request from the corresponding author.

## Supplementary Materials

The following supporting information can be downloaded at: https://www.mdpi.com/article/10.3390/vaccines10060925/s1, Figure S1: Flow cytometry gating strategy used to define CD4^+^ T effector and central memory cells, and the expression of transcription factors; Figure S2. Flow cytometry gating strategy used to define CD8^+^ T effector and central memory cells, and T-bet and granzyme B expression; Figure S3: Expression of transcription factors T-bet and RORχt in CD4^+^ T_EM_ cells of infected mice.; Figure S4: Expression of transcription factor T-bet and granzyme B in CD8^+^ T_EM_ cells of infected mice.
