# Supplementary material for: Protective Effect against Neosporosis Induced by Intranasal Immunization with Neospora caninum Membrane Antigens Plus Carbomer-Based Adjuvant
Source: Vaccines (Basel). 2022 Jun 10;10(6):925. doi: 10.3390/vaccines10060925 (PMC9230871; doi:10.3390/vaccines10060925)
Supplement: Supplementary file 1 [file vaccines-10-00925-s001.zip › vaccines-1731069-supplementary.pdf]

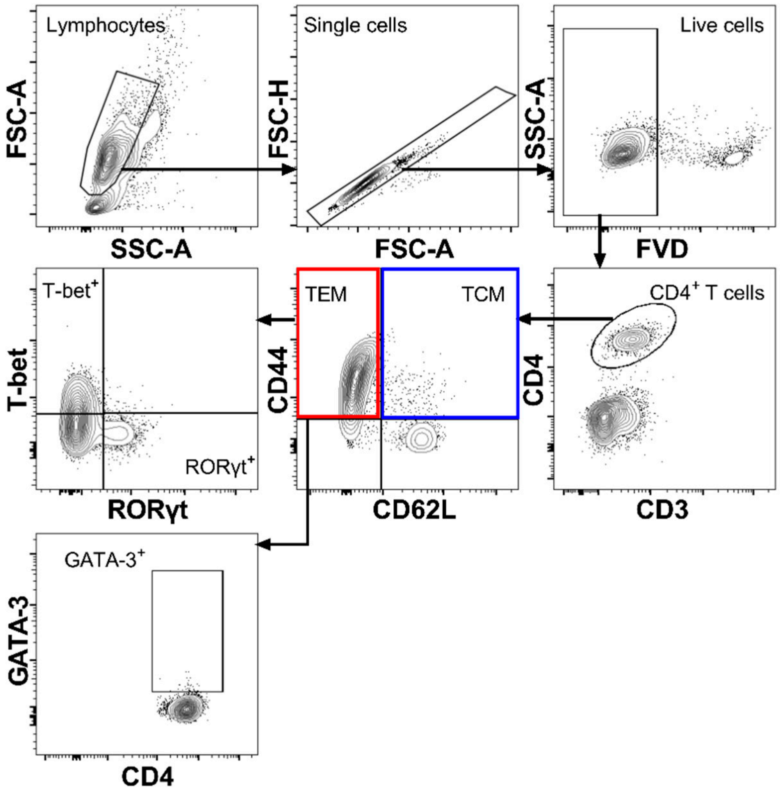

Figure S1 Flow cytometry gating strategy used to define CD4^{+} T effector and central memory cells and the expression of transcription factors. Lymphocytes were gated based on FSC and SSC parameters and doublets were excluded (Single cells) in FSC-H versus FSC-A plots. Viable cells (Live cells) were defined as fixable viability dye (FVD) negative cells. CD4 T cells were defined as CD3^{+}CD4^{+} and quadrants were set in contour plots, based on FMO stainings, to define effector memory (T_{EM}; CD44^{+}CD62L^{-}) and central memory (T_{CM}; CD44^{+}CD62L^{+}) cells. Gating of TEM cells expressing T-bet, RORγt and GATA-3 was defined based on FMO stainings. A similar analysis of the referred transcription factors was done gated in total CD44^{+} CD4^{+} T cells (T_{EM} plus T_{CM}). This is a representative example of lung cells isolated from a NcMP/CARB immunized mouse, 7 days i.p. post-infection with 1×10^{7} *N. caninum* tachyzoites.

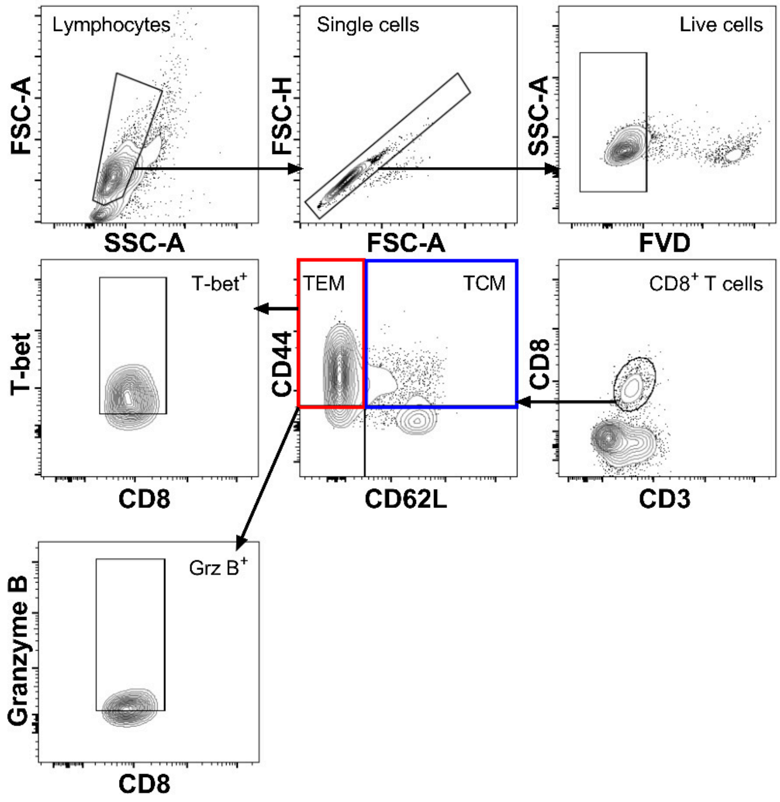

Figure S2 Flow cytometry gating strategy used to define $CD8^+$ T effector and central memory cells and the expression of T-bet and Granzyme B. Lymphocytes were gated based on FSC and SSC parameters and doublets were excluded (Single cells) in FSC-H versus FSC-A plots. Viable cells (Live cells) were defined as fixable viability dye (FVD) negative cells. $CD8^+$ T cells were defined as $CD3^+CD8^+$ and quadrants were set in contour plots, based on FMO stainings, to define effector memory (T_{EM}; $CD44^+CD62L^-$) and central memory (T_{CM}; $CD44^+CD62L^+$) cells. Gating of T_{EM} cells expressing T-bet (T-bet$^+$) or granzyme B (Grz B^+), was defined based on the respective FMO stainings. A similar analysis of T-bet- and granzyme B-expressing cells was done gated in total $CD44^+CD8^+$ T cells (T_{EM} plus T_{CM}). This is a representative example of lung cells isolated from a NcMP/CARB immunized mouse, 7 days i.p. post-infection with 1×10^7 *N. caninum* tachyzoites.

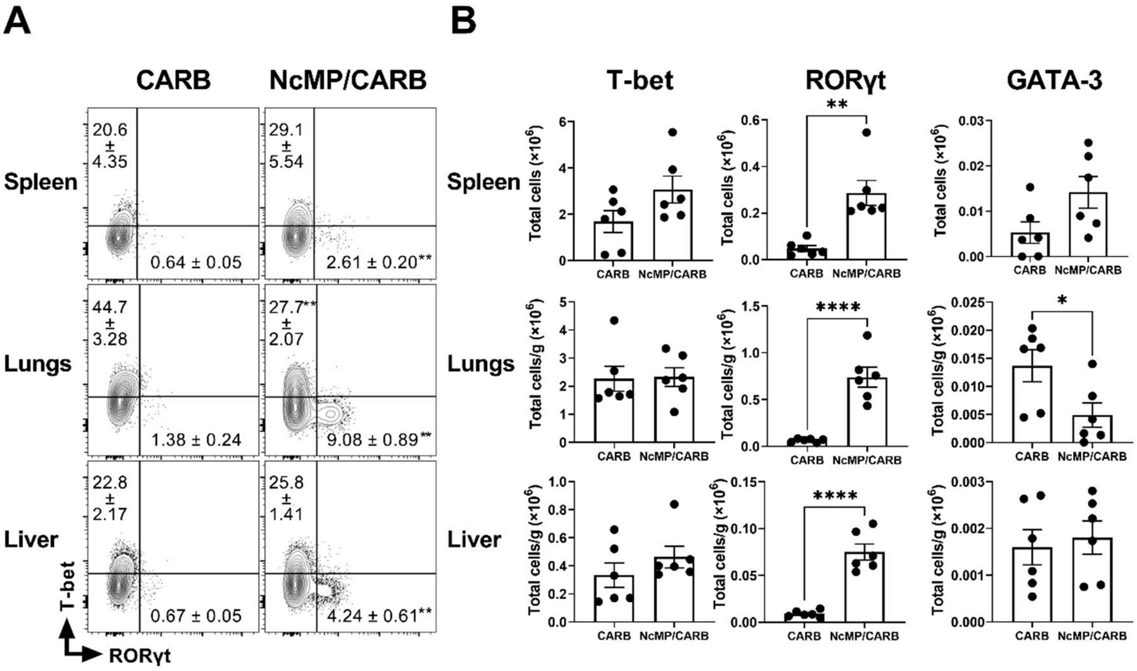

Figure S3: Expression of transcription factors T-bet and RORγt in CD4⁺ T$_{EM}$ cells of infected mice. (A) Representative contour-plot analysis of T-bet and RORγt expression in CD4⁺ T cells T-bet in the indicated organs of sham-immunized (CARB) or immunized (NcMp/CARB) mice, as indicated, 7 days after i.p. challenged with $1{\times}10^7$ *N. caninum* tachyzoites. Numbers within contour plots correspond to mean percentage values ± SEM of cells in the respective analysis regions. (B) Numbers of CD4⁺ T$_{EM}$ cells expressing T-bet, RORγt and GATA-3, in the indicated organs of infected mice of NcMP/CARB and CARB mouse groups. Results are representative of two independent experiments that yielded concordant results (n=6 group; **P* < 0.05; ***P* < 0.01; ****P* < 0.001; *****P* < 0.0001, Mann-Whitney).

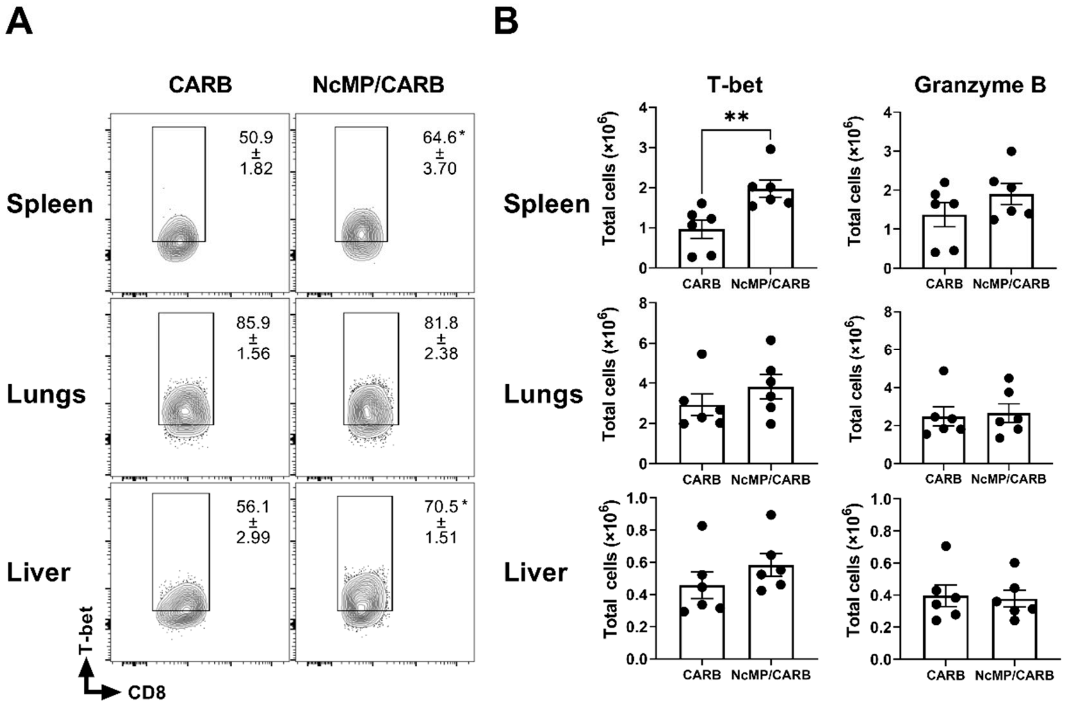

Figure S4 Expression of transcription factor T-bet and of granzyme B in CD8+ TEM cells of infected mice. **(A)** Representative contour-plot analysis of gated CD8+ TEM cells expressing T-bet in the indicated organs of sham-immunized (CARB) or immunized (NcMp/CARB) mice, as indicated, 7 days after i.p. challenged with 1×10^7 *N. caninum* tachyzoites. Numbers within contour plots correspond to mean percentage values ± SEM of T-bet expressing cells within the CD8+ T cell population. **(B)** Numbers of CD8+TEM T-bet+ or granzyme B+ cells in the indicated organs of immunized and sham-immunized mice. Results are representative of two independent experiments that yielded concordant results (n=6 group; *P< 0.05; **P< 0.01, Mann-Whitney).
